# On the Complexity of Visual Judgement of Kinship

**DOI:** 10.1177/2041669519841642

**Published:** 2019-05-06

**Authors:** Linda Brodo, Enrico Grosso

**Affiliations:** University of Sassari, Italy

**Keywords:** visual perception, face similarity, face dissimilarity, judgement of kinship

## Abstract

Discrimination of close relatives is a basic ability of humans, with demonstrated and important consequences in social and sexual behaviours. In this article, we investigate the visual judgement of kinship, that is the process of discriminating relatives based on visual cues and, in particular, on facial resemblance. Starting from triplets of face stimuli, we focus on a simple two-alternative forced choice protocol and we ask participants to evaluate kinship, similarity, or dissimilarity. Response times of the participants performing these visual judgements are recorded and further analysed. The analysis can also benefit from previous findings on the adopted face data set; in particular, results are compared with reference to an independently generated and statistically reliable similarity index, which is available for each possible considered pair of images. Our results confirm previous findings stating that kinship and similarity judgements are closely related and take longer, on average, than dissimilarity judgement. Moreover, they confirm that similarity and dissimilarity cannot be considered just as opposite concepts, and strongly support the existence of different pathways for similarity and dissimilarity judgements. Concerning kinship judgements, results confirm the assumption, inherent in previous models, of a close relationship between cues signalling for kinship and cues signalling for similarity but suggest the existence of a more complex process, where dissimilarity cues need to be explicitly included in order to model measured effects. Our results reinforce the idea that modulation mechanisms between similarity and dissimilarity measures could explain selective suppression or enhancement effects reported in previous works. A new framework is thus proposed hypothesising that kinship recognition is the result of a balanced evaluation of both similar or dissimilar pathways.

## Introduction

In recent years, research on face perception led to remarkable advances in the understanding of many different aspects of how faces are processed and memorised by the human brain (Little, Jones, & DeBruine, 2011). The impact of these findings will be significant on a wide range of disciplines and, notably, in the next generation of human-machine interfaces, as faces provide primary access to other people’s identity (Hole & Bourne, 2010) and act as drivers for important social interactions where relatedness (Alvergne et al., 2009; Kaminski, Dridi, Graff, & Gentaz, 2009), attractiveness (Perrett et al., 1998), mood (Ekman & Rosenberg, 2005), and other social cues play a crucial role (DeBruine, Jones, Little, & Perrett, 2008).

In this article, we focus on a specific and fundamental feature of face perception, that is the ability to recognise kinship from visual cues. This problem has been studied in the last decade (Dal Martello & Maloney, 2006; DeBruine et al., 2009; Lorusso, Brelstaff, Brodo, Lagorio, & Grosso, 2011) partly confirming the anecdotal belief of a close relation between facial resemblance and kinship perception. This ability seems common to all primates (Parr, Heintz, Lonsdorf, & Wroblewski, 2010) and seems also related to basic evolutionary behaviours. Interestingly, humans can reliably detect close relatives not only with respect to themselves but also between other individuals; this peculiar trait has been recently referred as allocentric kin recognition (Dal Martello, DeBruine, & Maloney, 2015).

**Figure 1.**
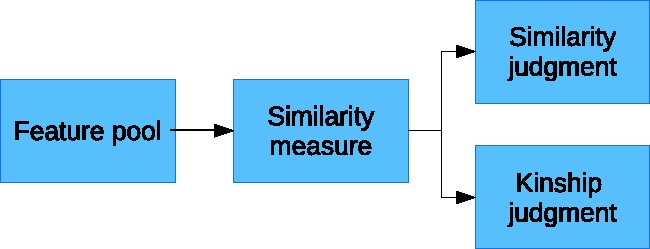
The TSO model proposed by Maloney and Dal Martello (2006).

A first attempt to model the process of kinship recognition and to capture the importance that similarity cues play in this process is due to Maloney and Dal Martello (Dal Martello & Maloney, 2006) who report a great accordance between similarity ratings assigned to pairs of related children by a first group of observers and the probability of judging those children as siblings by a second group of observers. To explain this result, they propose an original model, named Thresholded Similarity Observer (TSO), and based on the assumption that kinship and similarity judgements both rely on a common pool of facial resemblance cues (see Figure 1). In other words, they suggest a common visual processing pathway for both judgements of kinship and similarity and hypothesise a preliminary step of analysis in which the computation of a similarity measure, based on a pool of facial features, gives substantial support to the subsequent judgement of both similarity and kinship.

The TSO model has been recently investigated also by DeBruine and coworkers. They replicate the study of Dal Martello and Maloney with adult faces and report cases for which similarity ratings are not in accordance with previous results. They therefore conclude that similarity and kinship judgements cannot be considered synonyms, suggesting that “people use context-specific criteria for judging kinship and similarity in faces” (DeBruine et al., 2009, p. 42). From a more general perspective, note that results of DeBruine and colleagues seriously question the TSO model, as they suggest the existence of kinship cues that people can consciously or unconsciously ignore when performing similarity judgements of faces.

**Figure 2.**
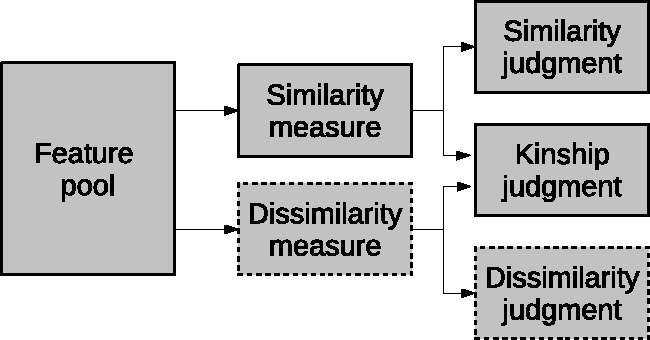
The model hypothesised by Lorusso et al. (2011).

Lorusso et al. (2011) report selective suppression or enhancement of the dissimilarity judgement in a study focused on the priming effect and explain these results hypothesising a separate pathway for judgement of dissimilarity (see Figure 2). Note, however, that while this extension of the TSO model allows to justify a selective suppression of the dissimilarity measure, without impacting the similarity measure, it does not clarify the real extent of the kinship judgement. In fact, the authors limit their discussion to a possible interaction between the specific task (for instance the kinship judgement) and the use of the available visual pools: “Perhaps a specific task instruction might trigger the preferential use of a feature pool associated with that task” (Lorusso et al., 2011, p. 1288).

The possible existence of different pathways in face perception, even though uncorrelated from a specific task like kin recognition, emerges from the work of Schwaninger, Lobmaier, Wallraven, and Collishaw (2009). They analyse the role of featural (local) and configural (global or structural) representations and conclude arguing about a two-route model of face processing and matching. A similar approach, from a more technical perspective, is taken by Tistarelli, Lagorio, and Grosso (2009); they focus in particular on the selection of relevant features and on the local or global matching strategy bringing to a single similarity score.

The importance of the spatial localisation of visual cues has been recently investigated also by Dal Martello et al. (2015). Differently from other judgements based on facial cues, they report that kinship judgements do not rely on cues disrupted by inversion. They explain this result suggesting a specific nature of the kinship judgement, related to the presence of local cues rather than holistic cues. The discussion on how recognition of faces is affected by the adoption of a configural representation becomes particularly interesting considering that face recognition in humans is successful over a variety of appearances due to light, pose, and external factors. To this respect, findings of Redfern and Benton (2017) suggest that expressions are part of facial representation, thus deeply coded in the basic mechanisms detecting and measuring similarities between faces.

Starting from this outline, our investigation focuses on the interrelation among judgements of kinship, similarity, and dissimilarity by measuring response times (RTs) for the three judgements. Are similarity and kinship analogous concepts? Are similarity and dissimilarity opposite concepts? And, more in general, are all these judgements based on common cues or common pathways?

The investigation moves from a first preliminary experiment, whereby an independent group of participants graded face-pairs on a 0-1 point scale: from entirely dissimilar to entirely similar. This experiment, which has been published in a previous research paper (Lorusso, Pulina, & Grosso, 2015), is briefly summarised in the next section for sake of completeness. A second experiment is then detailed where additional data are collected and the role of preliminary graded similarity is investigated with respect to judgements of kinship, similarity, and dissimilarity.

## Experiment 1—Calibration

The purpose of this section is to describe the selection and ratings of the stimuli to be used in Experiment 2. More in detail, a reliable perceived similarity index (PSI) is assigned to all the face-pairs, leveraging a procedure fairly complex for the statistics and the number of comparisons involved. As demonstrated in Lorusso et al. (2015), the PSI computed following this procedure is much more reliable than the PSI obtained by rating scales, an approach commonly used in psychophysics; moreover, it takes into account the context involved (i.e., the set of images used in the experiment) and some nonmetric behaviours that characterise the human judgement (e.g., the fact that comparing Face A to Face B is often different from comparing Face B to Face A, thus violating the axiom of symmetry that should be assumed for a metric model).

### Method

The computation of the PSI was performed for a pseudo-randomly generated set of 79 face-pairs, extracted from a data set of 54 face photos. For each face pair, a sequence of trials based on the two-alternative forced choice (2AFC) protocol was applied. Triplets used in the 2AFC trials were composed by the two images of the pair and a third image, randomly selected to cover the whole set of remaining 52 face-images.

#### Stimuli

**Figure 3.**
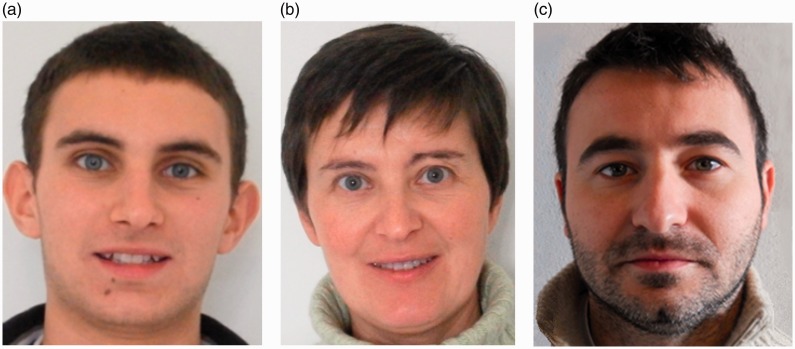
Three faces selected from the original data set of 54 images. Note that the central-left (a–b) and central-right (b–c) face pairs are characterised by very different similarity indexes: (a–b) very high PSI (0.98) and (b–c) very low PSI (0.08).

A homogeneous data set of 54 colour photographs, each depicting a face of a different person, has been carefully selected from photo albums of friends, without further processing. The manual selection took into account the resolution of the images (240 × 300 pixels at least), the expression (mostly spontaneous), and the background (conveniently homogeneous). Figure 3 shows some examples. Twenty-five male and 29 female faces were included in the data set, spanning an age range from 25 to 62 years. They were all of Caucasian appearance. Forty-four of the persons involved claimed to be in kinship relationship with others in the group. In total, 14 distinct family sets, each containing three or four members, were so declared. Each family set included different combinations of kinship pairs: biological siblings, father–son or daughter, and mother–son or daughter.

#### Participants

Participants in the experiment were 158 undergraduates and staff at the University of Sassari (84 females and 74 males), all reported to have normal or corrected-to-normal vision. Mean age of the participants was 24 years. The total number of participant has been chosen to guarantee a good level of confidence (>80%) on the result of the similarity judgement.

#### Equipment and detail of the procedure

Stimuli were presented on a computer monitor, screen resolution 1280 × 1024, refresh rate 60 Hz. Pictures were presented in triplets over a grey background. Triplets were composed by the two images of the selected face pair (from here on denoted as pair-reference, pair-candidate) and a varying third image (from here on denoted as varying candidate) taken randomly (but once at most) from the remaining images of the data set. Following this procedure, each selected face-pair gave origin to 52 different triplets. The pair-reference face was always positioned in the middle of the triplet; the pair-candidate face and the varying candidate were randomly positioned to the left or right side of the triplet. Participants had to make a forced choice between the candidate images (2AFC) indicating which of the two candidate faces looked more similar to the pair-reference face. Responses were collected by recording the mouse position at the click and the time elapsed since the presentation of the triplet. Subjects were given unlimited time to respond. The first three triplets presented per participant were considered as a training and were omitted from any subsequent data analysis.

Participants completed a randomised sequence of 2AFC trials for each selected face-pair; 79 face-pairs were selected in total by a pseudo-randomised procedure aimed at generating pairs having pair-reference images in common. To improve the statistical significance of the computed PSI, each single triplet was judged by six participants, thus each face-pair received six evaluations for each of the 52 AFC trials. In total, the experiment involved 4,108 trials (79 × 52) and 24,648 single judgements (79 × 52 × 6). Each of the 158 participants evaluated 156 triplets; the order of presentation of the triplets was organised randomically, but a manual, a posteriori, check was performed after the automatic selection of the triplets in order to guarantee the presence of different faces in two consecutive triplets and avoid memory effects.

### Results

**Figure 4.**
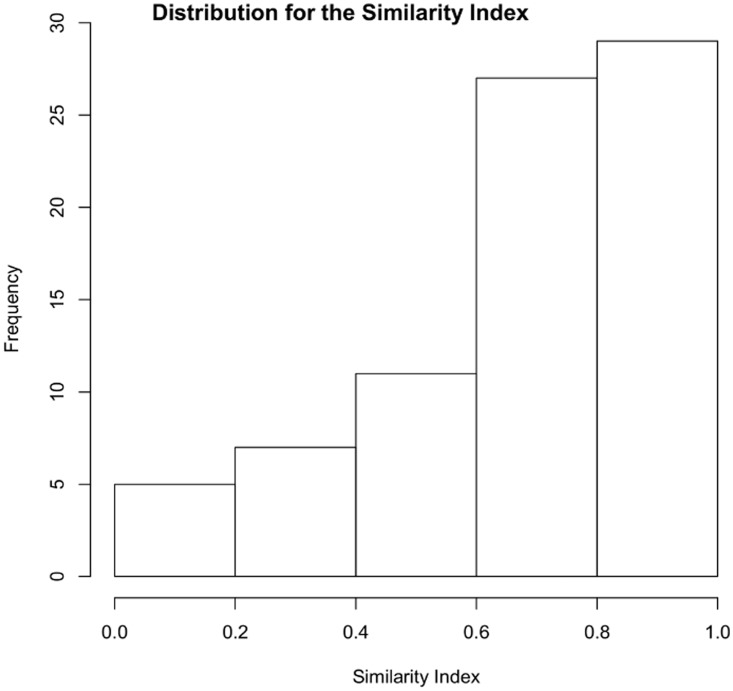
Distribution of the similarity index for the 79 pairs selected from the original data set of 54 images.

For a single trial, we considered significant the agreement on the similarity judgement, and therefore, the choice in favour of the pair candidate with respect to the varying-candidate image (or vice versa), only in presence of a concordant number of judgements equal to or greater than five out of six. As demonstrated in Lorusso et al. (2015), this result is statistically robust. Results of single trials were used to grade the complete set of *N* = 54 pictures *P_i_*. Proceeding through exhaustive comparisons, each selected pair (*P*_i_, *P*_j_), was compared with the remaining 52 pictures and the number of times *n*_p_ for which the similarity judgement between the pair-candidate and the pair-reference was greater than the similarity judgement between the varying-candidate and the pair-reference was counted. The ratio $S_{\text{ij}}=n_{\text{p}}/(N-2)$ is the PSI for pair (*P*_i_, *P*_j_). Figure 4 shows the distribution of measured PSI for the 79 pairs considered. The plot shows a significant level of agreement for most of the face-pairs, suggesting some kind of perceptual saturation; in other words, humans would tend to saturate rapidly the perceived similarity and locate similarity values more frequently at the top rather that at the bottom of the scale. On the other hand, however, a significant level of agreement could be simply related to the homogeneity of the data set chosen and the presence of kinship relations; in this case, an agreement on the topmost area of the scale would be an obvious consequence of the considered context (i.e., of the set of pictures).

Figure 3 gives the reader a more concrete example of the relationship between face-pairs and computed PSIs.

## Experiment 2—Similarity, Kinship, Dissimilarity

Starting from the results of the calibration experiment, the purpose of Experiment 2 was to understand the role of PSI in the judgement of similarity (S), kinship (K), and dissimilarity (D). The problem was investigated collecting a large amount of additional data and analysing the distribution of RTs with respect to the judgement; the effect of the PSI and the dependence on the stimulus type (i.e., the presence or absence of pairs related by kin) were also considered.

### Method

Based on the 2AFC protocol previously detailed, judgements were performed for a set of 69 triplets pseudo-randomly generated from the previous data set of 79 face pairs. For each face triplet, three trials based on the 2AFC protocol were performed; in the first trial, the participants were asked to judge about similarity, in the second and third trial about kinship and dissimilarity, respectively. Each participant always performed a single trial (similarity or kinship or dissimilarity).

#### Stimuli

Pictures used for the experiment are part of the data set used in the calibration experiment. From a total of 79 face-pairs for which the PSI was available, 69 triplets were pseudo-randomly generated taking into account the family relationship and the difference in PSI. Note that, from now on, the difference in PSI between face-pairs (left-central, right-central) will be denoted as PSI delta or PSID. In more detail, three simple constraints were imposed for the generation of the triplets:

1. large coverage of the available face-pairs, provided the existence of a common pair-reference image for each triplet;
2. large coverage of PSID values; and
3. large coverage of different types of stimulus (including people related or not-related by kin).

Due to the distribution of the PSI in the 79 face-pairs and to the kinship relationships involved in the original data set, the generation of the 69 triplets proved to be a very difficult optimisation task; starting from a repeated random selection of a first face-pair, the procedure tried to iteratively associate a second face-pair but always keeping in mind the cost of the constraints above and favouring minimal cost associations. All the constraints were supposed to have equal weight, in such a way to guarantee a quite equal distribution of the features characterising the stimuli.

**Figure 5.**
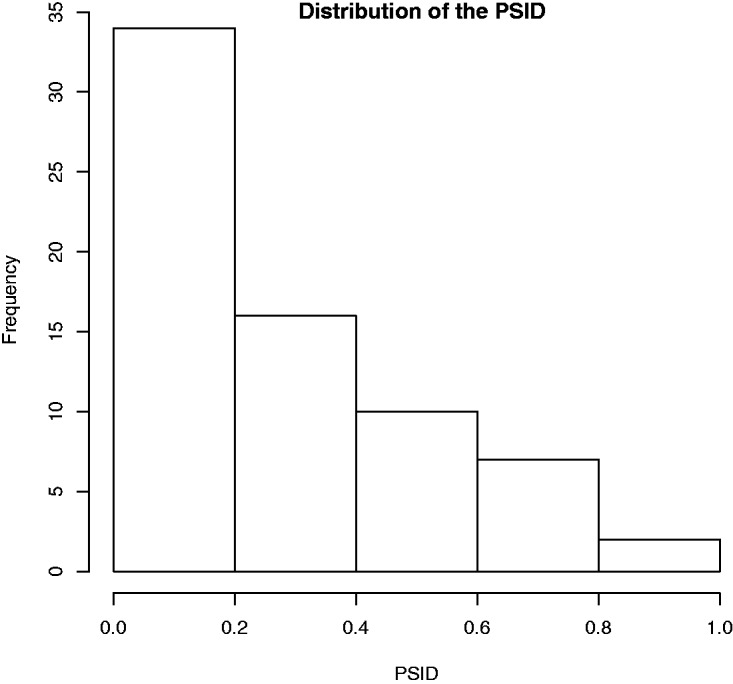
Distribution of the the difference in PSI (PSID) for the 69 face-triplets selected from the original data set of 54 images. PSID = perceived similarity index delta.

As shown in Figure 5, the final distribution of PSID for the triplets is far from equal; on the other hand, we judged this arrangement sufficiently representative due to the fact that it includes the full range of PSID values (from 0 to 1), giving the possibility to test the role of similarity on a large scale. Moreover, triplets include people related or not-related by kin; in particular three kinds of triplets can thus be considered:

- mixed (MIX)—where one of the two face pairs of the triplet belongs to people related by kin,
- nonmixed kin (KK)—where both face-pairs belong to people related by kin, and
- nonmixed non-kin (NK)— where both face-pairs belong to people not-related by kin.

#### Participants

Participants in the experiment were 35 undergraduates at the University of Sassari (19 females and 16 males), all reported to have normal or corrected-to-normal vision. Mean age of the participants was 23 years. As in the Experiment 1, the total number of participant was decided in order to guarantee a good level of confidence (>80%) and limited corresponding confidence intervals for all the possible outcomes of each trial.

#### Equipment and detail of the procedure

In this experiment, three were the judgement tasks involved: S, D, and K.

Stimuli were presented on a computer monitor, screen resolution 1280 × 1024, refresh rate 60 Hz. Each photo was digitised to 240 × 300 pixels, each depicting a face from the neck to the top of the head. Pictures were presented in triplets, displayed on a LCD screen, and viewed from a distance of approximately 50 cm. On screen, the three photos were separated by 1.5 cm upon a mid-grey background, with each photo having a height of 8.5 cm and a width of 6.5 cm. The location was a quiet room: Only the subject and the person illustrating the experiment were present. The latter remained at a distance so as not to disturb the subject. The experiments were carried out with artificial light to make it easier to see the screen. Participants had to make a forced choice between the candidate images (2AFC) indicating which of the two candidate faces looked the most similar (or dissimilar or related by kin) to the reference face. Subjects were given unlimited time to respond and the time elapsed since the presentation of the triplet was recorded. The first three triplets presented per participant were considered as a training and were omitted from any subsequent analysis. Data collected (responses and RTs) were further processed in order to discard meaningless behaviours of participants.

The 35 participants were divided in three groups, each assigned to one of the three tasks: We finally had 12 subjects assigned to Task S, 12 to Task D, and 11 to Task K; each of the three tasks was performed on the same 69 selected triplets. In summary, each single triplet involved three trials (S, D, and K), for a total number of 207 2AFC trials (69 + 69 + 69) and 2,415 single judgements (69× [12 + 12 + 11]). Each of the 35 participants evaluated only 69 triplets, expressing a single kind of judgement. As in the previous experiment, the order of presentation of the triplets was randomised and followed by a posteriori check, in such a way to guarantee the presence of different faces in two consecutive triplets.

### Results

#### Distribution of RTs

**Figure 6.**
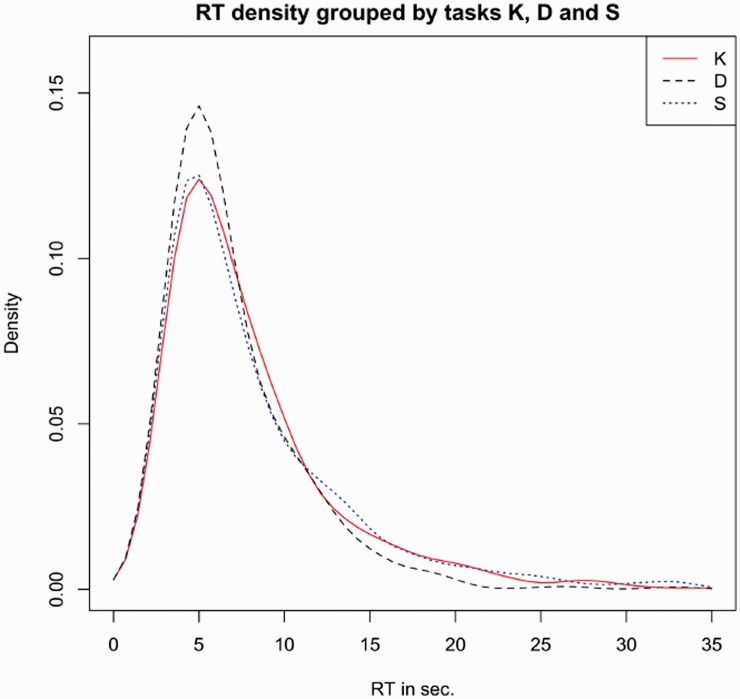
Distributions of the RTs by task. RT = response time.

The distribution of the RTs, grouped by task, is plotted in Figure 6. RTs are clearly condensed in the range [0–20] seconds with peaks around 6 seconds. Curves related to Task S and Task K appear very close; a little different trend characterises Task D.

At a first glance, distributions are similar in shape and not normal. The Shapiro–Wilk normality tests (Table 1) confirm this evidence for all the distributions.

**Table 1.** Result of the Shapiro–Wilk Normality Tests.

Set	*W*	*p*-value
S	0.733	2.2E−16
K	0.820	2.2E−16
D	0.548	2.2E−16

*Note.* S = similarity; K = kinship; D = dissimilarity.

Looking more carefully to the three distributions, Table 2 reports the medians and the tolerance of the RTs grouped by task. This result suggests that the dissimilarity judgement (D) somehow differs from similarity and kinship (S and K); in particular D seems to require a shorter execution time.

**Table 2.** Medians of the Response Times Grouped by Task.

			Tolerance α = .05, *P*=.99	
Task	*N*	Median RT	Lower side	Upper side
D	828	5.829	2.629	46.143
K	759	6.507	2.452	29.498
S	828	6.406	2.401	40.135

*Note.* For each group, the 95% tolerance interval with a coverage area of 99% is computed. RT = response time; S = similarity; K = kinship; D = dissimilarity.

To analytically test this evidence, we statistically compared the distributions of the RTs grouped by task. Taking into account the tolerance intervals of Table 2, outliers were first discarded removing RTs exceeding the tolerance limits. As shown in Table 3, this step caused the deletion of seven measures without remarkable effects on the median RTs of the three groups.

**Table 3.** Medians of the Response Times Grouped by Task After the Removal of the Outliers.

Task	*N*	Median RT
D	825	5.825
K	758	6.505
S	825	6.377

*Note.* RT = response time; S = similarity; K = kinship; D = dissimilarity.

We then applied the *Wilcoxon rank sum test*, with the usual null hypothesis *H*_0_ that the two populations under examination are the same. Table 4 shows the results of the comparison of the RTs for the three couples: (K–D), (S–D), and (S–K).

**Table 4.** Comparison of Distribution for the Response Times Grouped by Task, Using the Wilcoxon Test.

Tasks	*p-value*	Difference	Confidence interval
K vs. D	.0008	0.4856	[0.1079, 0.8715]
S vs. D	.0024	0.4319	[0.0640, 0.8129]
S vs. K	.8577	−0.0285	[−0.4446, 0.3854]

*Note.* S = similarity; K = kinship; D = dissimilarity.

In the cases (K–D) and (S–D), we can reject the null hypothesis, as the *p* value is small enough (less than .05) for both cases. For the case (S–K), we cannot reject the null hypothesis.

#### PSID versus RT

**Figure 7.**
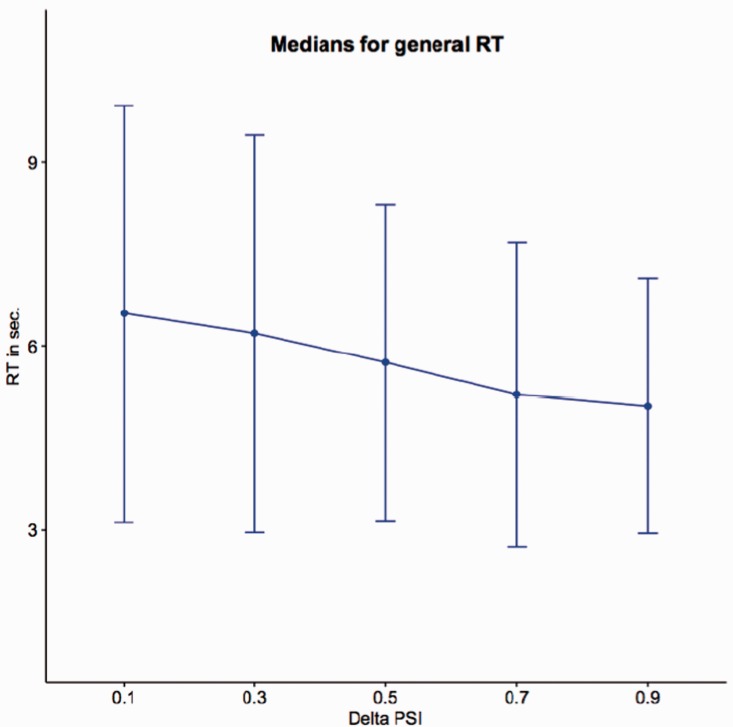
Medians of RTs with respect to PSID. Error bars are computed by standard Median Absolute Deviation (MAD). RT = response time.

To investigate the correlation between PSID and RTs, in Figure 7 we plotted the variation of the median RTs with respect to PSID. This plot gives a clear evidence of the relationship between the PSID and the time needed to accomplish the judgement: As expected, great differences in similarity correspond to shorter RTs confirming that judgements are in general more complex (and require more time) when faces are equally (or almost equally) similar. A statistical evidence of this correlation can be obtained by adopting a generalized linear mixed model (GLMM). This model can take into account both the nonnormal distribution of the measures and the random effects due to the subjects involved in the test. GLMM is first applied with the hypothesis of a simple fixed effect of the PSID (same dependency from PSID for all the subjects, fixed slope model) and then with the hypothesis that the effect of PSID might be different for different subjects (random slope model). To check the significance of the fixed effect with respect to potential artefactual effects, a third null model (no dependency from PSID) is also considered. In all cases, a log-normal family model is adopted for the distribution. Table 5 shows the results of the fixed slope model, which best fits the data. Note that the intercept estimate is 2.095, a value expressed in logarithmic scale and corresponding to 8.12 seconds (estimate for PSID = 0). In the same way, the slope of the PSID (−0.314) corresponds to a lowering of 1.37 seconds for PSID = 1, thus confirming the trend shown in Figure 7 for the medians.

**Table 5.** Application of the GLMM Model to the Response Times.

Fixed slope model—Random effects				
Groups	Name	Variation	*SD*	
Subject	(Intercept)	1.588	1.260	
Residual		20.567	4.535	
				
Fixed slope model—Fixed effects	Estimate	*SE*	*t*	pr(>|z|)
(Intercept)	2.09510	0.04845	43.24	<2*e*^−16^
PSID	−0.31469	0.04808	−6.55	5.94*e*^−11^

*Note.* Results of fixed slope model are shown. PSID = perceived similarity index delta; *SE* = standard error; *SD* = standard deviation.

**Figure 8.**
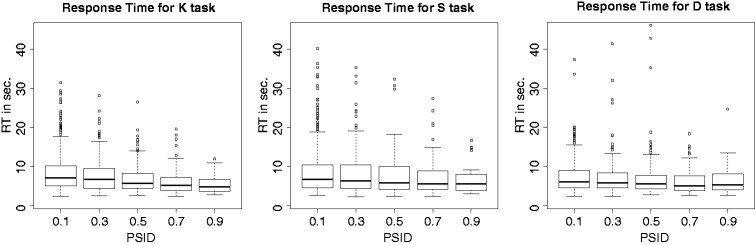
Distribution of the RTs with respect to PSID, subdivided by task. RT = response time.

Figure 8 gives some interesting details on how the tasks affect the general result of the previous Figure 7. In this case, boxplots are used in order to better show the distribution of the data. At a first glimpse, both S and K have similar behaviours, in accordance to the general plot of Figure 7; conversely, D seems less affected by variations of the PSID. To analytically investigate the effect of the task type, GLMM can be further applied, first under the hypothesis of a separated effect of PSID and task (no interaction) and then under the hypothesis of a mixed effect. The analysis of variance comparison of the two models demonstrates the existence of some mixed effects, and Table 6 shows in detail how the resulting GLMM model fits the data. Note that the first two lines of the table under the fixed effects section define the intercept and the slope of the measures belonging to the Group D. In summary, this result shows that both the intercept and the slope for Task D are lower than the corresponding values for the whole set of measures (Table 5). Following lines (Intercept S, PSID S; Intercept K, PSID K) show the variation of these values for the Groups S and K with respect do D. Note that variation in slope is highly significant for both S and K, thus being consistent with the differences arising from previous boxplots and the results of Table 4. No differences can be observed between S and K.

**Table 6.** Application of the GLMM Model to the Response Times

Fixed slope model—Random effects				
Groups	Name	Variation	*SD*	
Subject	(Intercept)	1.509	1.229	
Residual		20.459	4.523	
				
Fixed slope model—Fixed effects	Estimate	*SE*	*t*	pr(>|z|)
(Intercept) D	1.95046	0.08184	23.834	<2*e*^−16^
PSID D	−0.06902	0.08278	−0.834	0.404414
(Intercept) S	0.21739	0.11490	1.892	0.058487
(Intercept) K	0.20920	0.11751	1.780	0.075022
PSID S	−0.29751	0.11418	−2.606	0.009172
PSID K	−0.41778	0.12085	−3.457	0.000546

*Note.* The model assumes same dependency from PSID for all the subjects (fixed slope model) and takes into account the interaction between PSID and the task. PSID = perceived similarity index delta; S = similarity; K = kinship; D = dissimilarity; *SE* = standard error; *SD* = standard deviation.

#### Dependence of RTs on the stimulus

The dependence of RTs on the stimulus is shown by Table 7; for each of the three kinds of triplets considered, median RTs and related tolerances are reported.

**Table 7.** Median Times Grouped by Stimulus.

Stimulus	*N*	Median RT	Tolerance, α = .05, *P* = .99
KK	735	6.888	[2.485, 35.361]
NK	315	6.169	[2.383,46.143]
MIX	1,365	5.837	[2.615, 31.428]

*Note.* RT = response time; MIX = mixed; KK = nonmixed kin; NK = nonmixed non-kin.

In Table 8, we report the comparison of the RTs grouped by stimulus. We applied the *Wilcoxon rank sum test*, with the usual null hypothesis *H*_0_ that the two populations under examination are the same. The results show the comparison of the RTs for the three couples: (KK–NK), (KK–MIX), and (NK–MIX). In the first two cases, (KK–NK) and (KK–MIX), we can reject the null hypothesis, as the *p* value is small enough (less than .05) for both cases. For the case (NK–MIX), we cannot reject the null hypothesis.

**Table 8.** Comparison of Distribution for the Response Times Grouped by Task, Using the Wilcoxon Test.

Tasks	*p-value*	Difference	Confidence interval
KK vs. NK	.0012	0.6804	[0.2719, 1.1140]
KK vs. MIX	1.15e-09	0.8591	[0.5829,1.1402]
NK vs. MIX	.3099	0.1805	[−0.1673, 0.5229]

*Note.* MIX = mixed; KK = nonmixed kin; NK = nonmixed non-kin.

Here, some interesting points arise. First, it seems that kinship, when present in both pairs, has an influence on its own, as the distribution of the RTs for KK group is different from both NK and MIX. Second, looking at the groups KK and NK, a significant difference between kinship and similarity is observed. In fact, comparing the triplets KK (where kinship and similarity are present for both pairs of the triplets) and the triplets NK (where kinship is absent and similarity is present for both pairs), RTs suggest that the reported slowing effect could be specifically related to the presence of kinship information in the triplet.

The application of the GLMM model to this case (fixed slope model, interaction between PSID and the stimulus) partly confirms this hypothesis; as reported in Table 9, PSID MIX and PSID NK (last two rows) show a significant variation of the slope for the groups MIX and NK with respect to group KK. Statistically relevant differences between MIX and NK could not be observed.

**Table 9.** Application of the GLMM Model to the Response Times

Fixed slope model—Random effects				
Groups	Name	Variation	*SD*	
Subject	(Intercept)	1.574	1.254	
Residual		20.346	4.511	
				
Fixed slope model—Fixed effects	Estimate	*SE*	*t*	pr(>|z|)
(Intercept) KK	2.10292	0.05614	37.46	<2*e*^−16^
PSID KK	0.01061	0.12987	0.08	0.9349
(Intercept) MIX	−0.04378	0.03916	−1.12	0.2635
(Intercept) NK	−0.01915	0.05538	−0.35	0.7296
PSID MIX	−0.32198	0.14292	−2.25	0.0243
PSID NK	−0.40209	0.17764	−2.26	0.0236

*Note.* The model assumes same dependency from PSID for all the subjects (fixed slope model) and takes into account the interaction between PSID and the stimulus. PSID = perceived similarity index delta; *SE* = standard error; MIX = mixed; KK = nonmixed kin; NK = nonmixed non-kin; *SD* = standard deviation.

#### Mixed dependencies

Median times reported in Table 7 do not capture all the complexity of the results and, in particular, the earlier noticed specificity of the dissimilarity task. With the aim of better investigating this aspect with respect to the variation of the stimulus, we arranged Table 10, where median times are grouped first by stimulus and then by task.

**Table 10.** Medians of the Response Times Grouped by Stimulus and Then by Task.

Stimulus	Median RT for K	Tolerance α = .05, *P* = .99	Median RT for S	Tolerance α = .05, *P* = .99	Median RT for D	Tolerance α = .05, *P* = .99
KK	7.519	[2.623, 31.478]	6.933	[2.401, 35.361]	6.495	[2.431, 37.343]
NK	7.025	[2.383, 29.498]	6.469	[2.737, 33.193]	5.214	[2.429, 46.143]
MIX	5.995	[2.415, 28.976]	6.206	[2.286, 40.135]	5.768	[2.286, 40.135]

*Note.* RT = response time; MIX = mixed; KK = nonmixed kin; NK = nonmixed non-kin; S = similarity; K = kinship; D = dissimilarity.

Again, we observe that D is always the fastest judgement. Furthermore, it has a different behaviour with respect to S and K. In fact, while S and K follow the trend of Table 7 (both show their minimum for the MIX task), D has the minimum value for the NK stimulus.

**Figure 9.**
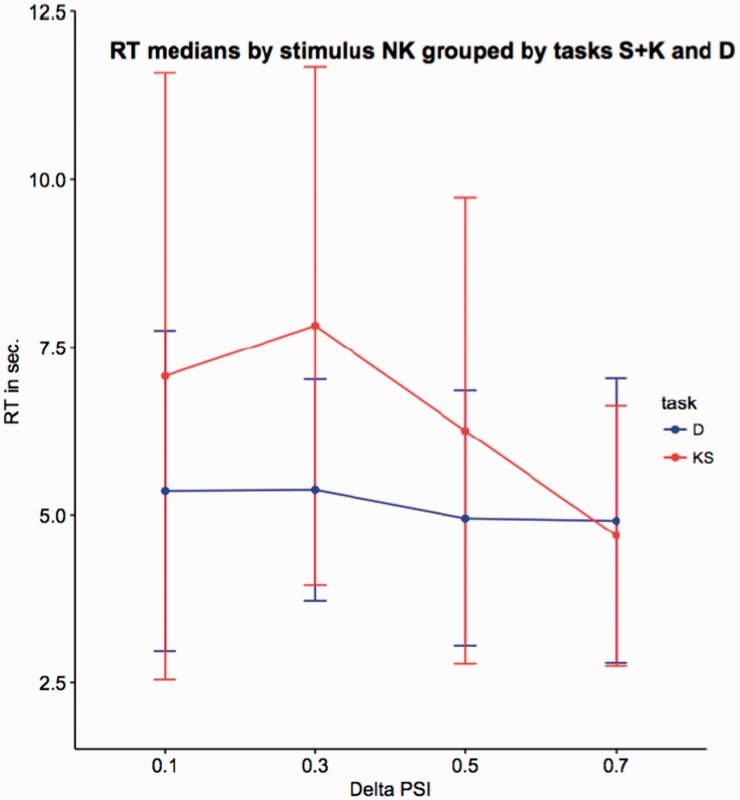
Medians RTs for the NK group of triplets, plotted with respect to PSID. In red, the times recorded for D task, and in blue, the times recorded for S and K. Error bars are computed by standard Median Absolute Deviation (MAD). RT = response time; PSI = perceived similarity index.

What happens for the NK group is of great interest due to the fact that pairs in the NK group certainly do not involve kinship information. We thus plot in Figure 9 the relationship between PSID and RT (as in Figure 7) but limiting the results to the NK group of triplets and distinguishing the trend of D with respect to S and K. The plot shows that for triplets not including kinship, the correlation between PSID and RT is really low; conversely, both tasks K and S show a trend more correlated to the similarity index, a result consistent with the plot of Figure 7.

**Figure 10.**
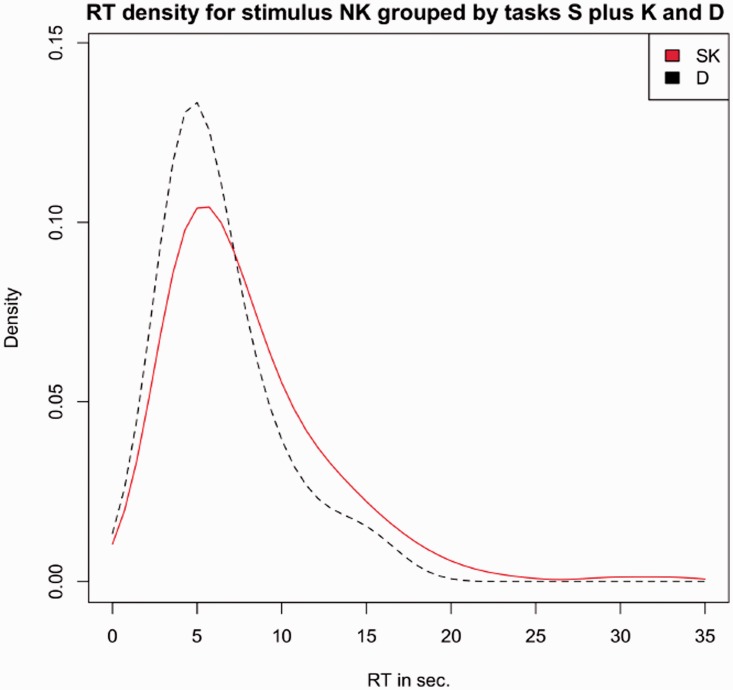
Distribution of the recorded RTs for the NK group of triplets of the mean RT with respect to PSID: In black, the times recorded for D task, and in red, the times recorded for S and K. RT = response time.

Figure 10 finally reports the same result in form of distribution functions. The shapes and the peaks of the two distributions for the NK group confirm that looking at faces not related by kin a clearer distinction between D and (S, K) tasks arises.

## Discussion

The results of the main experiment confirm and extend previous results, providing additional insights concerning the relation between kinship, similarity, and dissimilarity judgements.

A first important statement concerns the general timing of the judgements involved: as reported in Lorusso et al. (2011), Task D is always the fastest one and Task K is always the slowest. Our findings on RTs distributions confirm a distinction between D and K judgements but also show a distinction between D and S. In short, they **make it plausible the hypothesis of two different paths**, one of which reserved for D, the second probably related to S, or both S and K.

Looking at the results comparing PSID and RTs, the earlier statement takes shape further. An obvious remark is that the presence of similar pairs in the triplets makes judgements more complex (and slow). This holds for all types of judgements but it is particularly evident for S and K (see Figure 8), while D seems less affected by variations of the PSID. In our opinion, these evidences are consistent with the hypothesis of a double pathway (similarity – kinship or dissimilarity); moreover, they make plausible the existence of specific cues (fast) signalling for dissimilarity and substantially different from other cues (slow) signalling for similarity or kinship. The more dissimilar cues are present, the more the fast path takes strength and leads to a quick decision. One important consequence of this scenario is that **similarity and dissimilarity should not be considered just as opposite concepts** as they rely on different processing chains and probably on different cues. Moreover, as reported by Lorusso et al. (2011), a separate housing of the dissimilarity measure would help to explain suppression or enhancement mechanisms that appear to be closely related to our cultural and experiential stratification.

Concerning S and K judgements, results do not authorise to say much more. Our findings not only do not show a distinction between similarity and kinship but rather suggest some **close relation between kinship and similarity judgements** as they take, on average, similar times and they show quite similar behaviours with respect to PSID. In practice, kin cues could exist, and be considered together with similarity cues; on the other hand, kin cues might also not exist, following the TSO model (Dal Martello & Maloney, 2006), and resemblance cues might give support to both similarity and kinship judgements. However, the observed differences in RTs between KK and NK groups of triplets (last part of the experiment) help to better understand **the specificity of the kin judgement**.

This result is particularly interesting because it suggests us that kinship signals, if present, require additional time to be processed. This phenomenon should emerge in the case of mixed pairs (the MIX group, where the absence of such signals on a pair should speed up the judgement) and this should happen in particular for the K task (where the absence of kinship signals for a couple could lead immediately to the alternative choice). In fact, this is what happens in our experiment. Looking at Table 10, the median time for the MIX group in the K task falls under 6 seconds, at a level comparable to S and D tasks. Here it is worth noting that D again behaves differently from (S, K). In this case, kinship signals seem to disturb the dissimilarity task and the number of kin-related pairs involved in the judgement (one for MIX, two for KK) seems to be linked to the reported times. A possible explanation of this behaviour can rely on the fact that, measuring a type of information different from “resemblance,” D could be only weakly related to similarity by the aforementioned suppression or enhancement mechanism. In more detail, a strong activation of the slow path (due to resemblance cues existing in kin pairs) could suppress the fast path and somehow delay the final decision. This intuition is partly confirmed considering the last part of Experiment 2 and looking at the plot of Figure 9. For the NK group (which includes only pairs not related by kin), the variation of RTs with respect to PSID for Task D is really limited. This makes plausible the hypothesis of a fast path simply devoted to the dissimilarity task and almost totally independent from similarity cues.

How the fast path can contribute to similarity and kinship judgements is the last question arising in this discussion. Again with reference to Table 10, it is clear that task K somehow amplifies evidences recorded for S (long times for KK group, short times for MIX group); a possible explanation of this effect could be related to the fact that in some cases (namely KK and NK) **the final judgement of kinship could benefit from additional information coming from the evaluation of local differences**. In these cases, then, the long time recorded for Task K would derive from a “balanced” evaluation of the two paths.

In summary, the main points arising from the analysis of the experimental results are the following:

- The existence of a double pathway in visual judgement of kinship is highly plausible;
- The fast path of this double pathway could be related to cues signalling for dissimilarity; the slow path to cues signalling for similarity or kinship;
- There is no evidence of the existence of kin cues; however, the specificity and the complexity (additional with respect to similarity) of kin judgement clearly emerges;
- A plausible definition of the kin judgement could take into account both similarity and dissimilarity cues; in other words, kin cues would not exist per se but kin perception would exist in terms of a balanced evaluation of similar and dissimilar cues.

**Figure 11.**
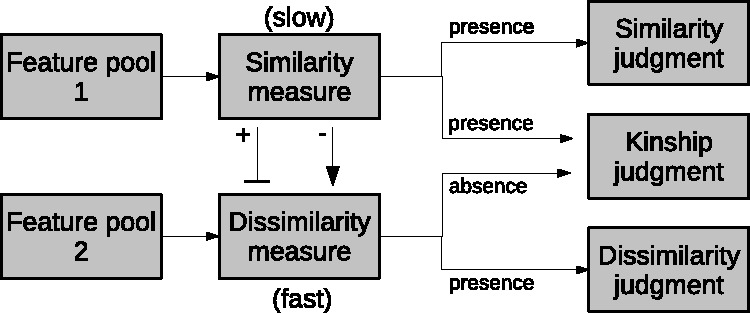
The proposed model.

Figure 11 shows a new framework which implements most of the above points. The main argument introduced by this framework is that S and K are not statistically different, and this evidence cannot justify the interpretation of kin recognition as an “additional level of analysis” based on a common pool of similarity cues. Our results overtake the anecdotal association of the concept of kinship with similarity and show that a different level of analysis, based on dissimilarity cues, might take place. Moreover, a suppression or enhancement process, probably related to cultural modulation, could intervene between two well-distinguished processing paths.

Our results strongly support the hypothesis of a double pathway (Schwaninger et al., 2009) where featural and configural signals are considered. Leveraging on the importance of the spatial localisation recently investigated by Dal Martello et al. (2015), the proposed model hypothesises that kinship, differently from other judgements based on facial cues, could take into account not only holistic (configural) cues but also the presence or absence of local (featural) cues. A straightforward way to implement this model is to assign to the two paths a different role: We argue in particular that similarity measure can play a configural role while a featural role would be better assigned to dissimilarity.

Future work will further investigate the relationship between visual and conceptual cues, like for instance the stereotypical association between concepts of similarity and kinship or similarity and nondissimilarity, trying to better define the amplitude and reach of the suppression or enhancement process. To this purpose, a very large number of participants (up to 30 for each task) will be involved, in such a way to increase the statistical coverage of the results. Effort will be also devoted to the refinement of the computational model proposed and to a novel definition of similarity or dissimilarity measures fully representing the configurational or local nature of the double pathway.
